# Atomistic Wear Mechanisms in Diamond: Effects of Surface Orientation, Stress, and Interaction with Adsorbed Molecules

**DOI:** 10.1021/acs.langmuir.3c01800

**Published:** 2023-09-27

**Authors:** Huong T. T. Ta, Nam V. Tran, M. C. Righi

**Affiliations:** † Department of Physics and Astronomy, University of Bologna, 40127 Bologna, Italy; ‡ School of Material Science and Engineering, Nanyang Technological University, 50 Nanyang Ave., 639798 Singapore

## Abstract

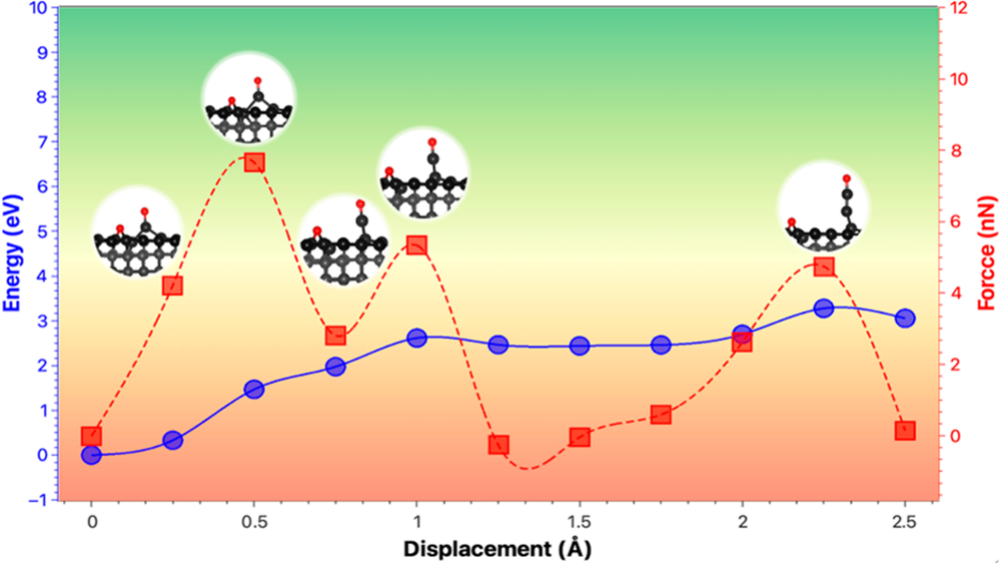

Despite its unrivaled hardness, diamond can be severely worn during the interaction with others, even softer materials. In this work, we calculate from first-principles the energy and forces necessary to induce the atomistic wear of diamond and compare them for different surface orientations and passivation by oxygen, hydrogen, and water fragments. The primary mechanism of wear is identified as the detachment of the carbon chains. This is particularly true for oxidized diamond and diamonds interacting with silica. A very interesting result concerns the role of stress, which reveals that compressive stresses can highly favor wear, making it even energetically favorable.

## 1 Introduction

The excellent wear resistance and ultralow friction make diamond an ideal choice for various tribological applications such as cutting tools, bearings, micro-electromechanical systems, and coatings.^1−3^ However, diamond is not completely inert to wear, especially under extreme conditions which experience high loads and a severe environment. The impact of the environment and/or the extreme working conditions result in material loss, high friction, and surface degradation and seriously undermine the functionality of the diamond-based devices.^4−7^ Understanding the wear mechanism on diamond surfaces is of utmost significance, not only for enhancing its applications but also for advancing material synthesis.

Several studies have described the chemical modifications of diamond surfaces under operating conditions, such as humid air, where atmospheric gases (H_2_, O_2_, and H_2_O) are present. The presence of H_2_ or H_2_O can create surface hydrogenation or hydroxylation, which have been shown to be beneficial for reducing friction and wear at the contacting interfaces.^8−11^ The primary mechanism of reduced friction is the surface passivation when carbon atoms from dangling bonds at the surface are terminated by H/OH. Recently, it has been shown that O_2_ dissociation is even kinetically and thermodynamically more favorable on diamond surfaces than H_2_ and H_2_O,^12^ making surface oxidation easier than hydrogenation or hydroxylation. The dissociative adsorption O_2_ not only alters diamond surface morphology but also modifies its electronic structure such as narrowing the band gap,^13^ changing the surface reactivity,^14^ or conductivity.^15^ In particular, O_2_ dissociation induces the breaking of surface dimer bonds, leading to the de-reconstruction on the C(100) surface.^16^ It has been found that the presence of oxidizing agents can promote the desorption of CO and CO_2_ molecules,^17,18^ which is the principle of oxidative etching and fabrication of diamond.^13^

Great effort has been devoted to understanding the atomic mechanisms of chemical and mechanical polishing processes of diamond surfaces,^18−21^ and several mechanisms have been proposed. In particular, the sp^3^-to-sp^2^ transformation has been proposed to explain the experimental observation of amorphous carbon and amorphous wear particles.^22^ By MD simulations, it was shown that C–C bond breaking on the C(110) surface starts at the weak C–C bonds which connect the C–C zigzag chain to the bulk diamond when sliding against silica. In this case, the C–C bond breaking can be initiated through strong Si–C and O–C bonds where Si and O play as mechanically supported pilot atoms.^23,24^ This event was not observed when sliding against silicon,^24^ highlighting the role of counter surfaces on the diamond polishing process. By a tight-binding quantum chemical molecular dynamics simulation, Kubo et al. proposed two mechanisms of wear, i.e., atom-by-atom and sheet-by-sheet removal of diamond polishing in the presence of OH radicals acting as an oxidizing agent.^21^ In H_2_O_2_ solution, it has been reported that carbon atoms can react with decomposed −H, −OH, and −O, leading to the formation of C–H, C–OH, and C–O. By MD simulations, the authors found that C atoms can be removed in the forms of CO, CO_2_, or carbon chains as a result of the combination of chemical and mechanical effects.^18^ Another aspect is the dependency of wear on surface orientations.^20,22,25^ While C(111) is reported as the hardest plane to be polished,^22^ C(001) and C(110) are more vulnerable and easier to be worn.^25^ This point has been proven and clearly shown on clean surfaces. Nevertheless, the effects of oxidation and the adsorption of atmospheric species on diamond polishing remain unclear. A detailed investigation of the effect of adsorbed O and other chemical groups on diamond polishing is of significance for its widespread applications in practical conditions.

In this study, we present a new computational procedure grounded in first-principles calculations to systematically characterize different atomistic wear mechanisms and quantify their associated energy costs and forces. By applying this methodology, we investigate the influence of surface orientation, namely, the C(001), C(110), and C(111) surfaces of diamond, as well as the adsorption of O, H, and OH species, interaction with silica, and surface stress (compression and expansion) on the wear mechanism. Our findings reveal that the adsorption of an oxygen (O) atom in the ketone configuration facilitates the detachment of carbon atoms from the diamond surface, thus initiating the wear process. Additionally, we uncover a wear mechanism on the diamond surface involving the formation of extended carbon chains. The C(110) diamond surface was identified as the surface that was most susceptible to wear in our study. Conversely, the adsorption of other species has a limited impact on the wear mechanism. Furthermore, we demonstrate that the initiation of carbon atom wear on the diamond surface necessitates the presence of a robust bond from the molecule or counter surface, such as a double bond or two single bonds. These insights shed light on the underlying mechanisms governing wear phenomena in the context of diamond surfaces and offer valuable implications for understanding and controlling wear processes at the atomic level.

## 2 Simulation Methods

In this research, density functional theory (DFT) simulations were performed using the Quantum ESPRESSO software.^26^ The exchange-correlation term was described using the generalized gradient approximation (GGA) as parametrized by Perdew–Burke–Ernzerhof.^27^ To account for the long-range van der Waals interactions, a semiempirical correction by Grimme (D2) was used.^28,29^ This combination of GGA-PBE and D2 has been found to provide an optimal balance between accuracy and computational cost, as indicated by previous studies.^30,31^ The convergence threshold was set at 10^–4^ Ry for the total energy and 10^–3^ Ry/bohr for the ionic forces. The self-consistent electronic (SCF) loop was set to converge at 10^–6^ Ry. Spin polarization was included in all calculations, as the presence of surface dangling bonds and dissociated molecules could result in magnetization in the system.

In this study, three distinct diamond surfaces were evaluated, namely, the C(110), the dimer-reconstructed C(001) and the Pandey-reconstructed C(111) surfaces. The simulations utilized a large orthorhombic supercell with a minimum lateral dimension of 8.74 Å in order to minimize interaction with periodic replicas. The C(110) and C(001) surfaces were modeled by using a 4 × 4 in-plane supercell, while the C(111) surface was simulated by using a 4 × 3 in-plane slab. The slab thickness used to model the C(110) corresponds to 7 atomic layers. Meanwhile, slabs of 8 and 10 atomic layers have been used for the C(111) and C(001) surfaces, respectively. The thickness values were selected based on previous studies,^32−35^ for obtaining accurate structural and energetic properties. A 20 Å vacuum region was included in the supercell to separate each slab from its periodic replica along the [001] direction. All simulations employed a plane wave cutoff of 30 Ry and a Monkhorst–Pack (MP) k-point mesh of 2 × 2 × 1. The cutoff energy, k-point mesh, and vacuum thickness were tested to ensure that the energy error was less than 3 meV/atom.

## 3 Results and Discussion

### 3.1 Wear of O-Containing Diamond Surfaces

**Figure 1:**
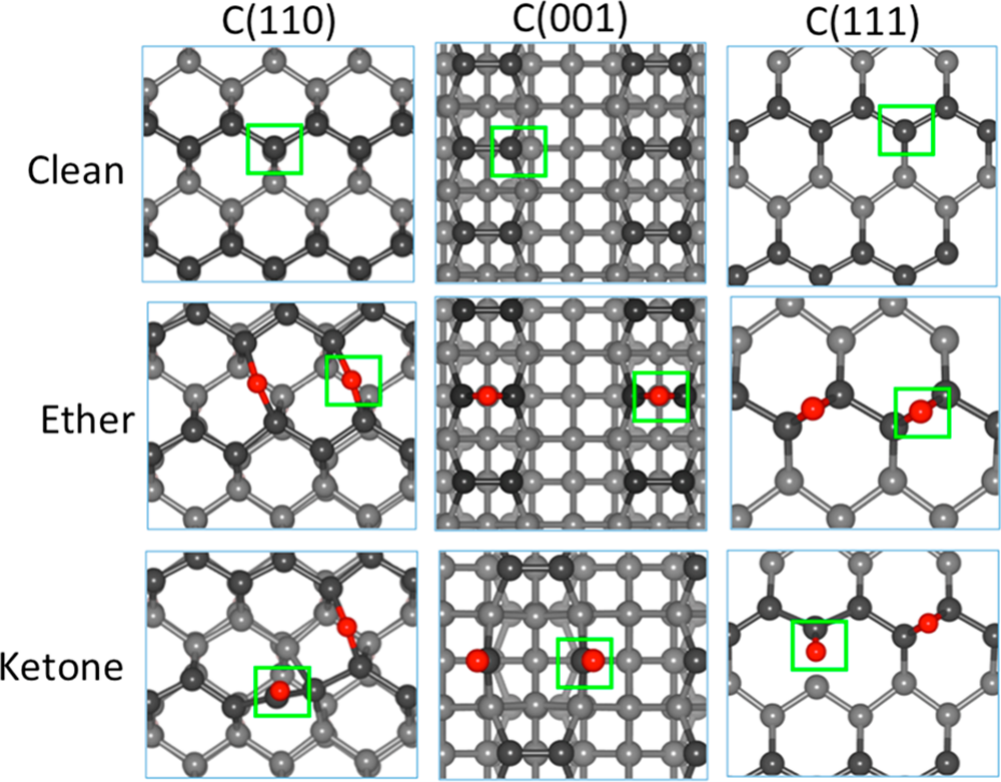
Initial structures for clean diamond surfaces and the dissociative adsorption of oxygen on the three diamond surfaces. C (gray) and O (red). The bold color is to show the carbon atoms at the top layer. The green boxes show the constrained atoms.

We first investigate the effect of oxygen adsorption on the wear mechanism of diamond surfaces including C(110), C(001), and C(111)-Pandey compared with that of the clean ones. The initial structures for the calculations are presented in Figure 1. The dissociative adsorptions of the oxygen molecule are considered with both ether (oxygen atoms form C–O–C bonds with the carbon atoms of diamond surfaces) and ketone (oxygen atoms form a double bond with a single carbon atom of diamond surfaces C=O) configurations (Figure 1). Previous works showed that ketone configurations are more stable for the C(110) and C(001) at low adsorption coverage.^13^ Meanwhile, the configuration is highly unfavorable for the C(111) reconstructed surface.^12^

**Figure 2:**
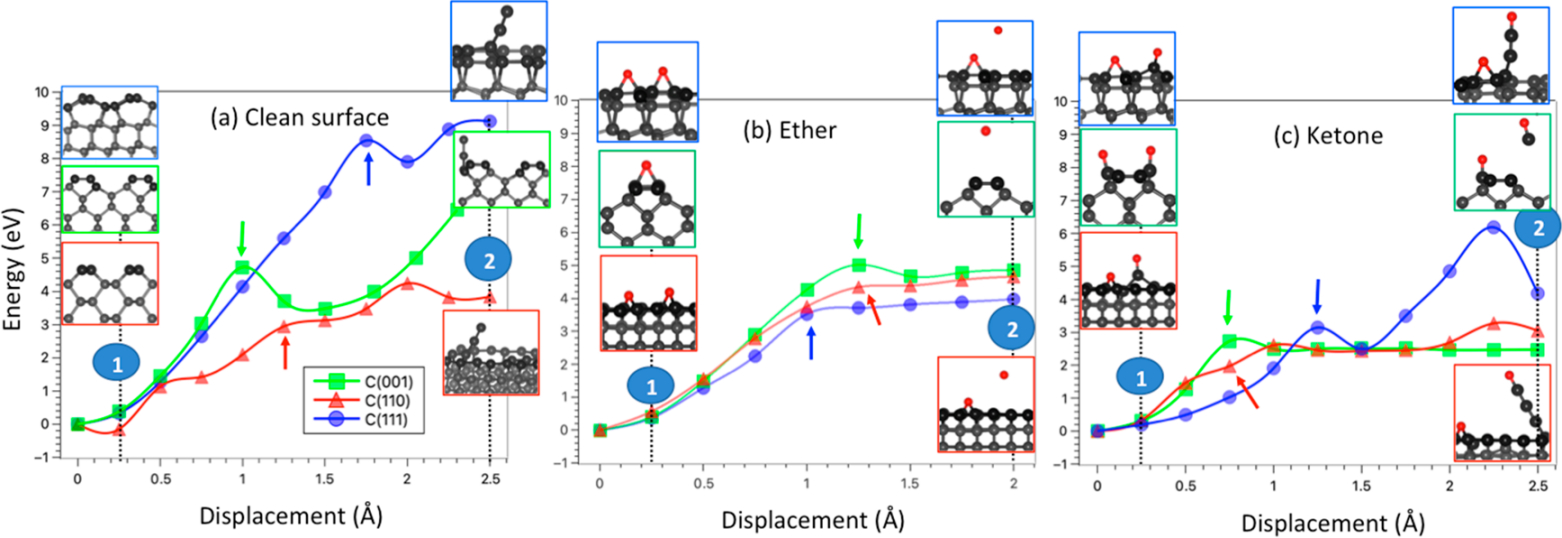
Wear of clean (a), ether (b), and ketone (c) containing diamond surfaces.

In practical working conditions, diamond surfaces are in contact with a counter body. The extreme condition results in the bond formation between the oxygen or unpassivated carbon and other atoms of the counter surface.^9,36^ The rubbing of the two surfaces leads to the stretching of the interfacial bonds and lifting of the oxygen atom or the underlying carbon off the surface. In an ideal scenario and to compare the wear mechanisms induced on the clean and different passivation of diamond surfaces, we mimic the effect of wear by pulling one atom (the adsorbed O atom or the C atom for the case of clean surface) along the vertical direction. In particular, for each step, the oxygen atom is displaced by 0.25 Å, followed by an atomic relaxation with the position of the O atom fixed along the *z* direction. The pulling energy is calculated by subtracting to the energy of the system where one atom is displaced out of its equilibrium position the energy at equilibrium. Meanwhile, the pulling force is the residual force along the vertical direction on the constrained atom, which is calculated by the Quantum Espresso program. In Figure 2a the energy increase due to the displacement of the pulled atom is reported as a function of the atom–surface distance (the corresponding forces are reported in Figure S1). The arrows indicate the first atom’s detachment from the surface. We show that very high pulling energies are required to detach the carbon atom from the clean surface. In particular, pulling energies of 2.95, 4.73, and 8.54 eV are required to remove the first carbon atom from the C(110), C(001), and C(111) clean surfaces, respectively. The high extreme pulling energy of C(111) is consistent with previous experimental and theoretical studies indicating the surface is very stable.^12^ On the other hand, the lower stability of C(110), which has higher surface energy than the other two surfaces, explains the reason for the lower energy cost for the wear processes. Another interesting observation common to all the considered surfaces is that the removal of carbon atoms is likely in the form a carbon chain, which is consistent with what found in diamond polishing.^18^ It is worth mentioning that in the counter surface environment, the carbon chain with carbon dangling bonds can react with other species, promoting a permanent loss of carbon atoms of the surface. Thus, the carbon chain established from our DFT calculations can be considered as the precursor for more complex tribochemical reactions with counter materials or environmental species.

The adsorption of oxygen in the ether configuration is unlikely to cause wear of the diamond surfaces through the detachment of the oxygen atoms, which occurs without causing any deformation of the underlying carbon–carbon bonds (Figure 2b). The energy trend is similar for all three surfaces, in which the system energy increases as the oxygen atom is pulled up and becomes flat when the oxygen atom is detached from the surface. On the contrary, the pulling up of oxygen atoms in the carbonyl (ketone) group can lead to the detachment of carbon atoms from the surfaces. We found that the wear mechanism depends on the surface orientation. For the C(110) and C(111) surfaces that terminate with zigzag chains, the displacement of oxygen atoms can cause progressive detachment of the surface chains, which is consistent with experimental findings on wear regime of diamond.^23,37^ Both the force (Figure S1) and the energy (Figure 2c) are much higher for the C(111) surface, as this surface is highly stable.^12^ Meanwhile, the wear on the C(001) surface will likely form a CO molecule rather than a carbon chain (Figure 2c). Compared to the case of the clean surface, the presence of oxygen atom in the ketone configuration helps to reduce the energy costs for carbon detachment. In particular, the pulling energies required to detach the first carbon atom from the surface are reduced to 1.97, 2.51, and 3.13 eV for the C(110), C(001), and C(111) surfaces. Among the three surface directions, the C(110) is the most easily wearable because the associated energy cost and restoring forces (Figure S1) are lower than for the other surfaces. It is worth noting that when the diamond surface is in contact with a counter surface, the pulling process proceeds through an interfacial bond connecting the two surfaces. Thus, the O atom of the C=O bond is bonded to other atoms, and a double C=O bond cannot be established. However, the strength of the C=O bond can be resembled when the carbon atom is bonded to two single bonds, as in the bidentate configuration of the silicate. More details about this adsorbed configuration are discussed in Section 3.2.

**Figure 3:**
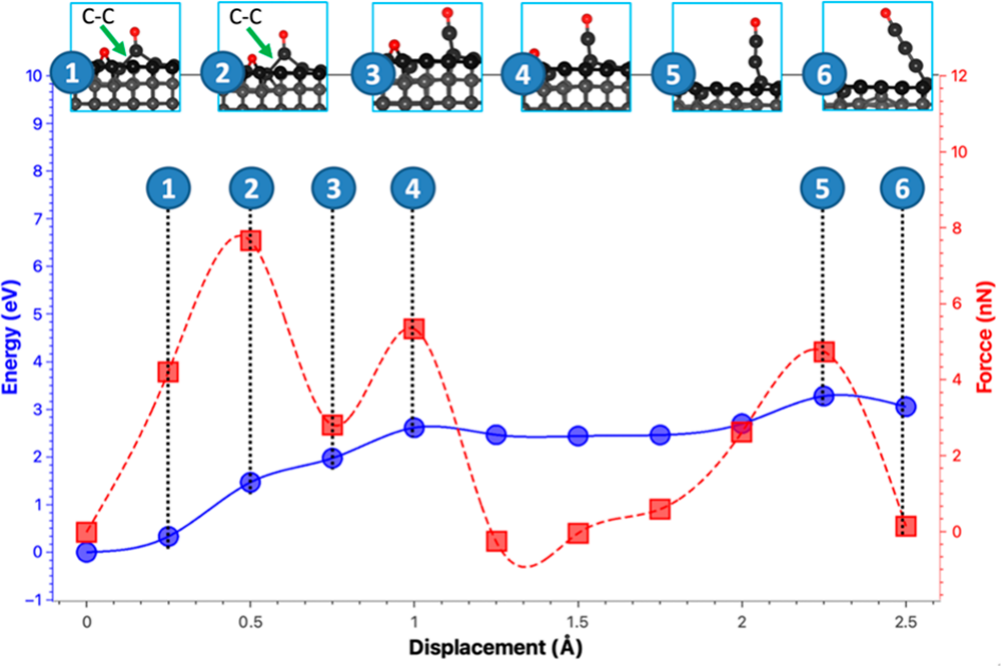
Energy (blue) and force (red) evolution under detachment of a carbon chain on the C(110) surface during the wear process.

As our calculation shows that C(110) is the easiest surface orientation to be polished, further calculations of wear mechanisms and effects of adsorbed species will focus on this surface. First, the energy changes and detailed atomistic mechanism of carbon detachment from the C(110) surface are depicted in Figure 3. When the oxygen atom is displaced from the surface (step 1 → 2), the two C–C bonds are stressed, causing an increase in the energy and restoring force acting on the displaced atom. After a C–C bond is broken (indicated by the green arrow), the force decreases to a lower value. If the displacement of the O atom is continued, the restoring force on the O atom increases again (step 3 → 4) until the second C–C bond with the surface is broken. The dissociation of one of the two C–C bonds is a prerequisite for the establishment of a carbon chain. Primarily, it facilitates upward traction of the chain and detachment of the carbon atom from the substrate. Second, the cleavage of one C–C bond results in the transformation of the remaining C–C bond into a double bond. The presence of these double bonds is essential to enduring the mechanical forces exerted during the pulling process. As this mechanism is repeated, the carbon chain becomes longer and longer. It is also worth mentioning that the longer the carbon chain, the longer the displacement that it takes for the force to increase. The result is due to the elastic properties of the carbon chain that as a collection of springs added in series becomes less stiff as a new C–C bond is incorporated into the chain.

**Figure 4:**
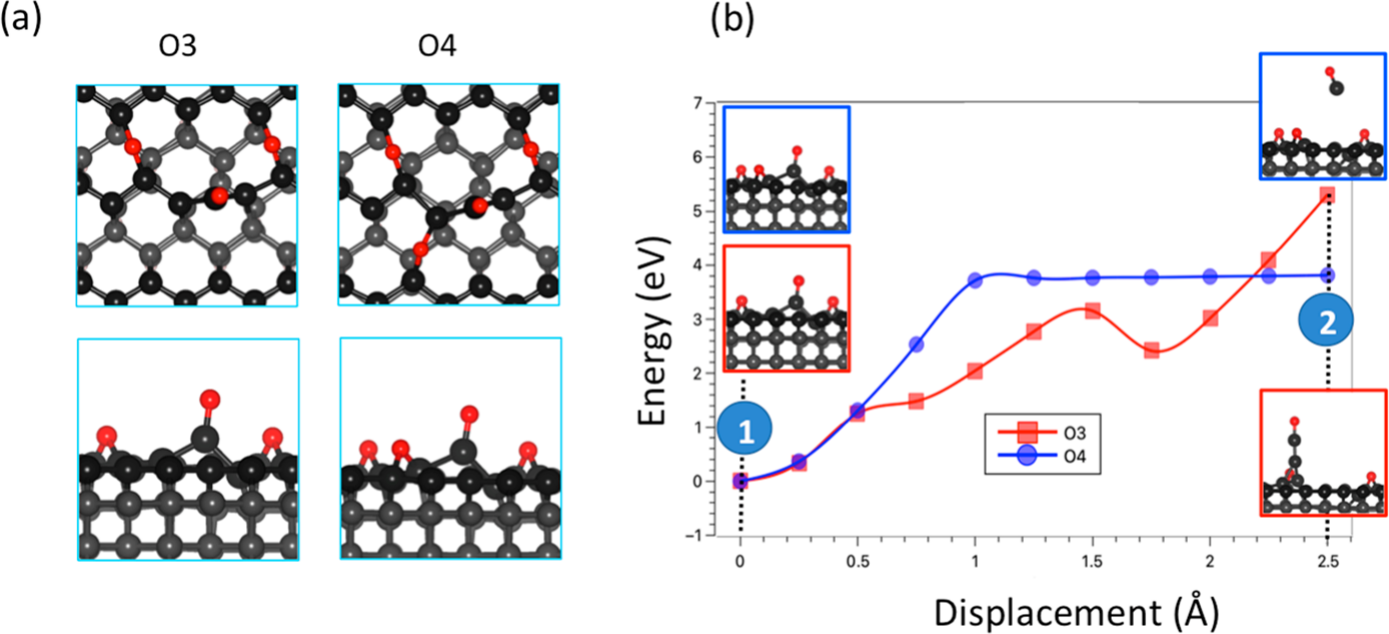
Top and lateral views of the O adatom arrangements considered for the C(110) surface (a). Energy change during the out-of-plane displacement of the O atom belonging to the ketone group (b).

We examined the effects of the presence of other O atoms adsorbed in the surrounding area of the ketone group (Figures 4a and S2). The previous calculation shows that only ketone configuration could facilitate the formation of carbon chain. Consequently, we initiated our investigation by considering the C(110) structure, which incorporates the ketone configuration, as depicted in Figure 1. Subsequently, we introduce additional oxygen atoms one by one close to the ketone site, which is chosen on the basis of the previous calculation by Chaudhuri et al.^38^ We found that when an additional O adatom (O3, red) is adsorbed far from the ketone group, the wear mechanism and the associated energy behave similar to what observed when only two O atoms are adsorbed (Figure 3), i.e., a carbon chain is formed upon the displacement of the O atom forming a double bond with the first detached carbon. On the contrary, when an additional O adatom (O4, blue) forms a bond with a first neighboring site of the ketone group, the detachment of the carbon chain is inhibited and only the C atom belonging to the ketone group is detached through the formation of a CO molecule (Figure 4), as observed in the case of the C(001) surface. This could be because the increase in the density of adsorbed oxygen follows by the reduction of bond strength of C–C bonds involving the O adsorption. The C–C(−O) bond is broken to create a CO molecule rather than a carbon chain. As a result, the energy shows a different trend with respect to that shown in Figure 3. The result suggested that the wear mechanism as well as the length of the C chain is affected by the adsorption of other species around the ketone group.

**Figure 5:**
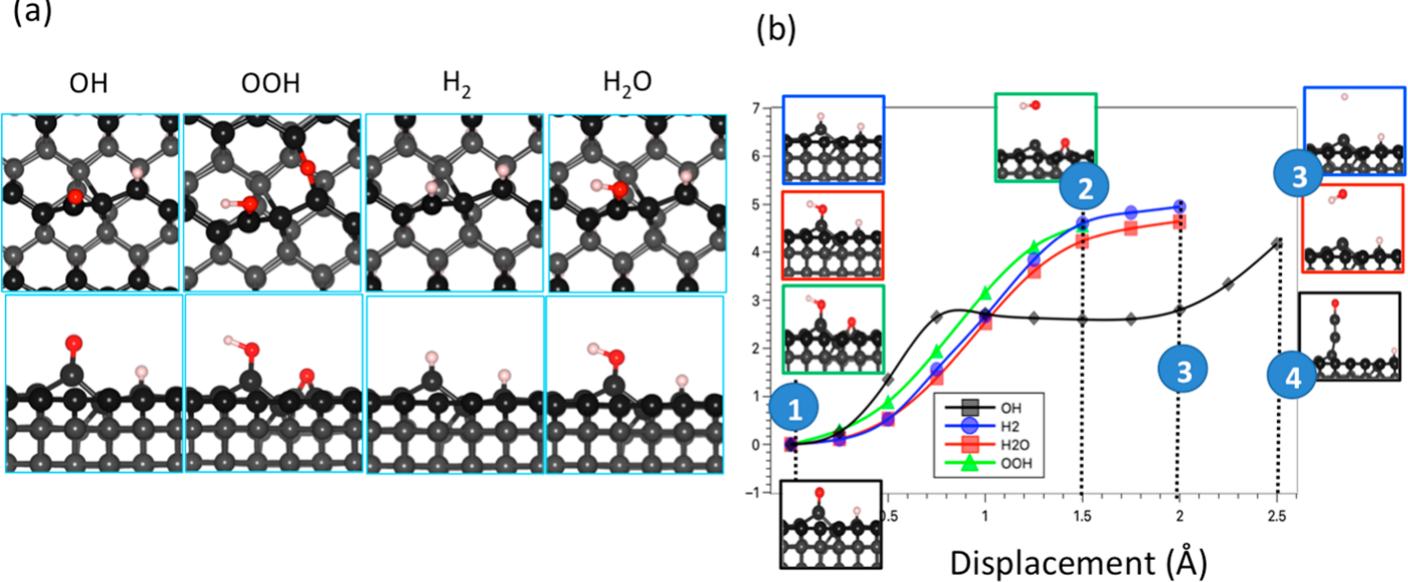
Top and lateral views of the chemical groups adsorbed on the C(110) surface (a). Energy during the out-of-plane displacement of the H, OH, and O groups (b). C (gray), O (red), and H (white).

We further extend the study of the wear mechanism on the C(110) surface by considering the presence of different chemical species and dissociated molecules including OH, OOH, H_2_, and H_2_O, as shown in Figure 5a. These species are highly present in the working environment as products of the dissociative adsorption of atmospheric gases or precursor molecules used for diamond growth.^39−41^ Starting with the most vulnerable configuration to wear (ketone configuration in Figure 1), we straightforwardly substitute the oxygen atom with different species and atoms. We found that when the oxygen atom in the ketone group is replaced by a H atom or OH group, wear cannot be initiated by the out-of-plane displacement of these groups: the displaced atoms are detached from the surface without causing any deformation for the C–C bonds of the substrate. The energy (Figure 5b) and force (Figure S3) trends are similar for H/OH. This could be because these groups form single bonds with the substrate. These single bonds are weaker than the O=C double bond presented in the ketone configuration, so the C atom dragged by the pulled atom cannot withstand the restoring force from the substrate. Owing to the analogous zigzag arrangements of carbon atoms atop both the C(110) and C(111) surfaces, a comparable wear mechanism is anticipated to emerge for these surfaces upon interactions with different adsorption species. On the other hand, we believe that the wear mechanism on the C(001) surface, characterized by the formation of CO molecules, is unlikely to undergo significant alteration upon the adsorption of alternative species. This is due to the fact that (1) the carbon atoms on top of C(001) surface do not adopt a connected zigzag chain configuration. Consequently, they are prone to dissociation upon application of an upward force. (2) The adsorption of other species will weaken the C=C double bonds. This outcome results from the necessity for carbon atoms to share electrons to form a covalent bond with other species (H or OH). As a result, the bond strength is weakened and will facilitate more the formation of CO molecule. Second, if the O forms a double bond with the C atom, it could reduce the strength of other C–C bonds as more electrons are shared to form the O=C double bond. As shown in Figure 5b (black line), the energy cost necessary to pull out of the surface the carbon chain terminating with an O atom (resulting, e.g., from the dissociation of a OH group) is much lower than the energy cost to detach the OH or H groups from the surface. Thus, the pulling of H or OH groups is not possible to promote the detachment of any C atom from the surface. Therefore, we conclude that the dragging specie to initiate wear should form a double bond with the displaced carbon atom, as stronger than the restoring C–C bonds.

In summary, the formation of wear and its mechanisms of diamond depend greatly on surface orientations and adsorbed species. Among the three surfaces studied, the C(110) surface was identified as the most susceptible surface to wear at both the clean and the O-adsorbed structures. Under oxygen adsorption, only the ketone configuration can initiate wear on diamond surfaces through CO desorption or carbon chain. In addition, the contribution of adsorbed species such as H/OH/OOH, and oxygen coadsorption on mechanism of wear was also investigated. Although H/OH/OOH adsorption shows a minor effect on diamond wear, the coadsorption of oxygen atoms can alter the wear mechanism induced by ketone configuration from carbon chain to CO desorption. This is the synergistic effect from multiple C–O bond formation, resulting in a reduction of C–C bond strength and facilitating C–C bond rupture. The calculation results highlight the intricate interplay between surface properties and adsorbed species on the wear mechanisms of diamond.

### 3.2 Wear Due to the Interaction with a Silicate

**Figure 6:**
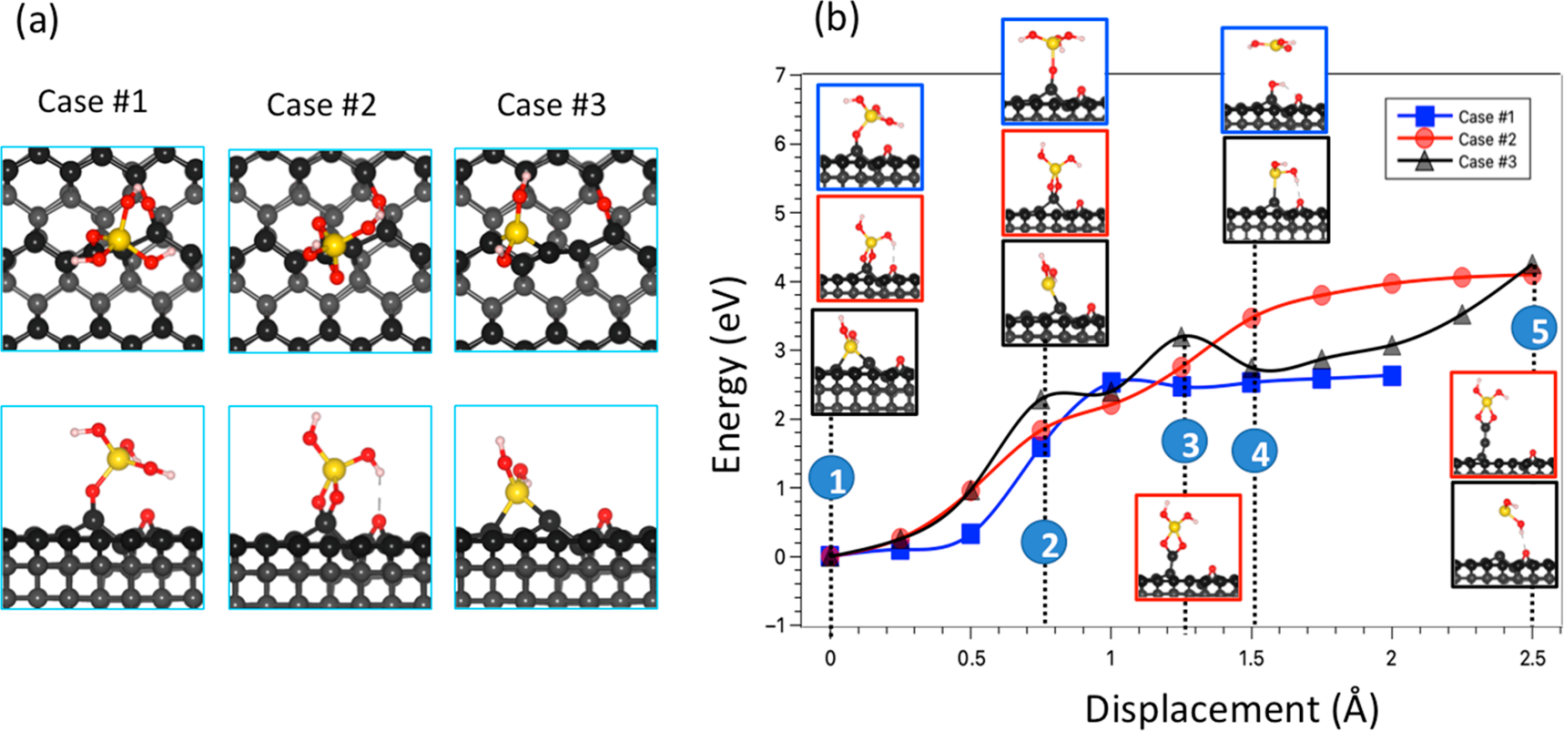
Different initial adsorption configurations of a silicate cluster on the C(110) surface (a). Wear mechanism caused by the detachment of the silicate cluster on the C(110) surface (b). C (gray), O (red), H (white), and Si (yellow).

Experimental studies have indicated that diamond can be worn when the sliding against silicate.^5,25,42^ The initiation of wear is associated with the formation of chemical bonds across the interface.^9,36^ In such a case, the local environment where Si–O–C bond formation occurs becomes extremely useful to examine atomistic mechanisms of wear. During sliding, the relative movement of silicate against diamond leads to the stretching of the Si–O–C bonds to promote wear.^24^ The impact of the counter surface sliding thus can be directed to the movement of Si atom lifting up the oxygen atom through the Si–O–C connection. To investigate this situation, we adopt a simplified model containing a silicate cluster interacting with the C(110) surface, which was found to be the most easily wearable. We consider three different adsorption configurations of the silicate cluster shown in Figure 6. In all of the initial configurations, the silicate cluster is positioned on top of an out-of-plane carbon atom. As shown in Figure 6b, when the silicate cluster bonds to the surface through single Si–O–C bonds (one in case #1 in blue and two in case #3 in black), the Si–O bonds broken after the Si displacement, leading to a sudden drop of force (Figure S4).

On the other hand, when the silicate cluster is bonded with the surface through two Si–O–C single bonds involving the same C atom, known as bidentate structure, the pulling up of the silicate fragment can lead to the formation of a C chain as shown in Figure 6b (case #2 in red). This mechanism is similar to that observed when the O atom of the ketone O=C group is pulled, as described in Section 3.1, and we observe a similar trend in the energy (Figure 6b) and force (Figure S4) curves. Particularly, the force on the Si atom peaks each time the carbon chain is stressed and drops suddenly when the C–C underlying bond is broken, and the longer the carbon chain, the longer the displacement that it takes for the force to rise. The result suggested that the two single bonds involving the same C atom can pull strongly enough to cause its detachment from the surface, as observed in the C=O double bond.

In comparison with the O-adsorbed C(110) surface, the single Si–O bond plays a role similar to that of the ether configuration while the bidentate structure can be a resemblance of the ketone configuration. In this context, the wear mechanisms are deeply governed by the bond strength of the bond that the pulled atoms form with carbon. Thus, the interactions of single and double Si–O bonds with the carbon atoms belonging to the other surfaces of diamond C(001) and C(111) can result in similar effects to those described for the ether and ketone adsorption configurations of oxygen on these two surfaces.

### 3.3 Effect of Stress on the Wear Mechanism

**Figure 7:**
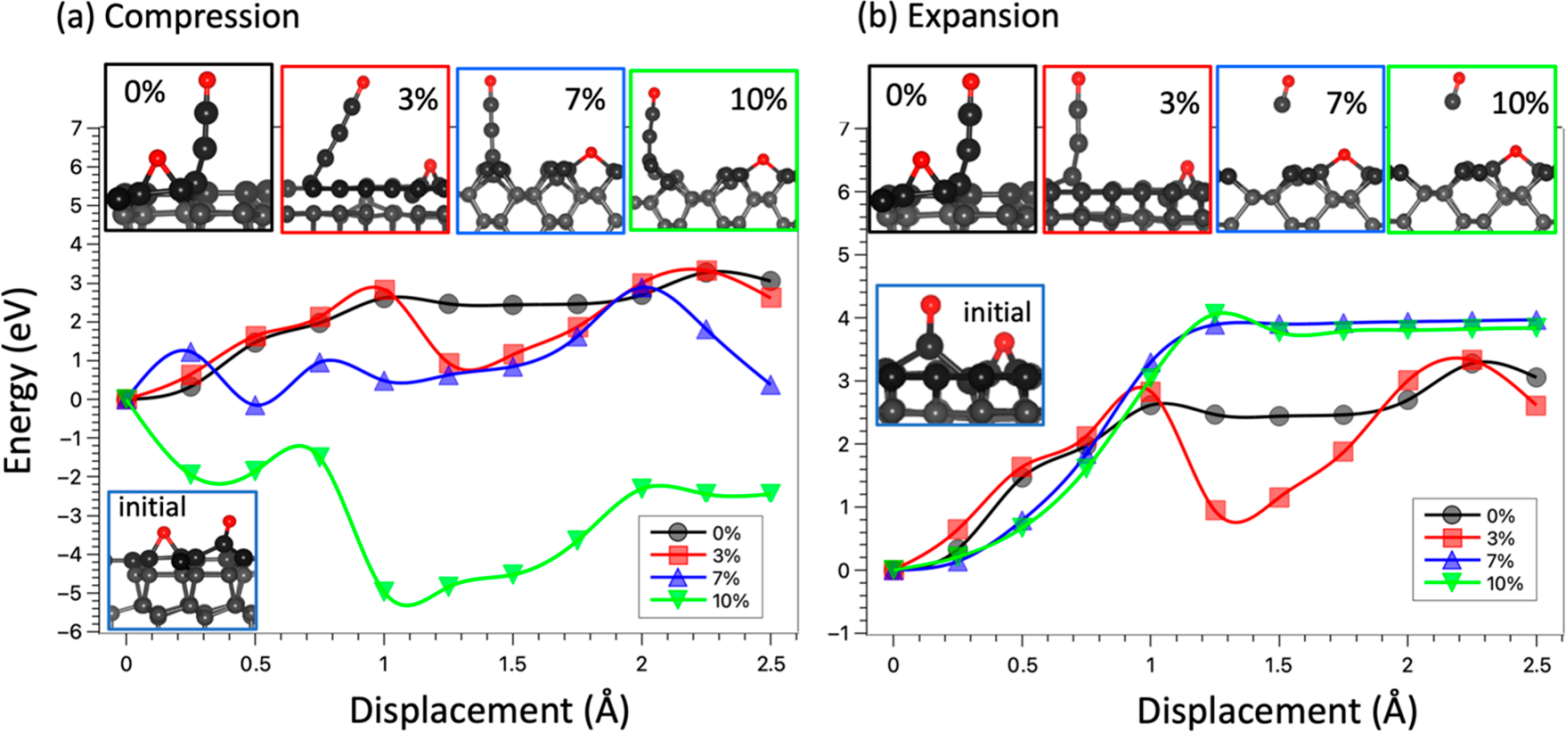
Wear mechanism caused by the adsorption of oxygen on the C(110) surface under different stress levels.

In tribological conditions, the surface can be subjected to stress as a result of sliding, loads, and preparation of the material,^23,37,43,44^ which can lead to the peeling-off of carbon atoms from the diamond surface.^45^ Consequently, it is crucial to investigate the impact of stress on the wear mechanism of diamond surfaces. In the present study, stress is introduced by varying the lateral size of the supercell with four stress levels considered, including 0%, 3%, 7%, and 10%. Such high values of stress can be experienced at sharp asperity–asperity contacts where the local load and temperature are extremely high, and deformation takes place.^46^ Furthermore, the application of extreme stresses, especially 7% and 10%, aims to accelerate significant changes within the system. The objective is to understand the influence of stress on the atomistic wear mechanisms of diamond. The evolution of energy and diamond structures subjected to stress are presented in Figure 7.

In the case of compression (as depicted in Figure 7a and the forces in Figure S5), our findings indicate that the wear mechanism remains unchanged with the detachment of oxygen atoms leading to the formation of long carbon chains. However, the stress can significantly alter the energy cost of wear. Specifically, the energy required to detach the oxygen atom decreases as the surface is compressed, particularly under high-stress conditions (7% and 10%). Interestingly, when the surface is compressed at 10%, it becomes energetically favorable for carbon atoms to detach from the surface, as shown in Figure 7a. Therefore, our result suggests that under extremely high load, the wear on the diamond surface can become severe.

On the other hand, the wear mechanism can undergo significant alteration when the surface is subjected to expansion. As depicted in Figure 7b, the formation of long C chains remains the predominant wear mechanism at lower stress levels (0% and 3%). However, when the stress level is increased, it fosters the formation of CO molecules rather than long C chains. Our findings reveal that both energy and force are augmented as the wear mechanism involves the formation of CO molecules. Moreover, there is a negligible difference observed between stress levels of 7% and 10%. Compared with the compression, the expansion promotes the formation of CO molecules rather than carbon chains. This is because the C–C bond distance is shortened under compression and elongated under expansion. The bond elongation makes the C–C bond weaker and thus unable to withstand the pulling forces as in the compressed case. Thus, the C–C bond at the surface is detached, and a CO molecule is produced. Furthermore, the expansion leads to the reduction of the diamond density, making it unlikely to release its atoms as a chain, as found for the compression.

## 4 Conclusions

In conclusion, the atomistic mechanisms of diamond wear are investigated by first-principles calculations considering different surface orientations, adsorbates, and stress levels in the carbon film. Our findings can be summarized as follows.

- The primary mechanism of wear involves the detachment of carbon chains from the surface. The C(110) surface is the most easily worn. Meanwhile the Pandey-reconstructed C(111) surface shows the best resistance to wear.
- The restoring force on the displaced C atom gradually increases until a C–C bond breaks on the diamond surface. A carbon chain is then formed by repeating this mechanism. Interestingly, the elastic properties of the formed chain resemble those of springs added in series: the longer the carbon chain, the lower the restoring force and energy increase.
- In the presence of O, H, and OH adsorbates, the formation of a chain pulled by a ketone, C=O, requires lower energy than on the clean diamond surfaces. On the contrary, the single adsorbate–C bonds turn out to be less strong than the surface C–C bonds; thus, only the pulled adsorbate is detached, without causing any distortion of the underlying carbon surface.
- When the adsorbate is a silicate, our results indicate that single Si–O–C or Si–O bonds are not enough to break C–C bonds on the diamond surface. Two Si–O–C bonds involving the same C atom in a bidentate structure are instead able to promote the C detachment from the surface and initiate the wear process through chain formation.

The stress level present on the diamond surface can significantly impact its wear resistance. A compressive stress decreases the energy cost for the detachment of carbon atoms, making wear an energetically favorable process under highly stressed conditions. Conversly, when the surface is subjected to expansion, the wear mechanism of carbon chain formation is inhibited, and removal of C atoms occurs through the formation of CO molecules at a high energy cost.

## Supporting Information Available

Evolution of forces during the pulling processes of clean and O-adsorbed diamond (110), (001), and (111) surfaces, force changes during the detachment of O/H/OH, and a silicate cluster on C(110) surface, force changes by the adsorption of oxygen on C(110) surface under different stress levels (PDF)
