# Supplementary material for: Atomistic Wear Mechanisms in Diamond: Effects of Surface Orientation, Stress, and Interaction with Adsorbed Molecules
Source: Langmuir. 2023 Sep 27;39(40):14396–403. doi: 10.1021/acs.langmuir.3c01800 (PMC10569040; doi:10.1021/acs.langmuir.3c01800)
Supplement: Supplementary file 1 — la3c01800_si_001.pdf [file la3c01800_si_001.pdf]

Supporting Information

Atomistic Wear Mechanisms in Diamond: Effects of Surface Orientation, Stress, and Interaction with Adsorbed Molecules

Huong T. T. Ta[1], Nam V. Tran[2,*], M. C. Righi[1,*]

[1] Department of Physics and Astronomy, University of Bologna, 40127 Bologna, Italy

[2] School of Material Science and Engineering, Nanyang Technological University, 50 Nanyang Ave, 639798, Singapore

Corresponding Author

M.C. Righi – E-Mail: clelia.righi@unibo.it, Department of Physics and Astronomy, University of Bologna, 40127 Bologna, Italy.

Nam V. Tran– E-Mail: vannam.tran@ntu.edu.sg, School of Material Science and Engineering, Nanyang Technological University, 50 Nanyang Ave, 639798, Singapore.

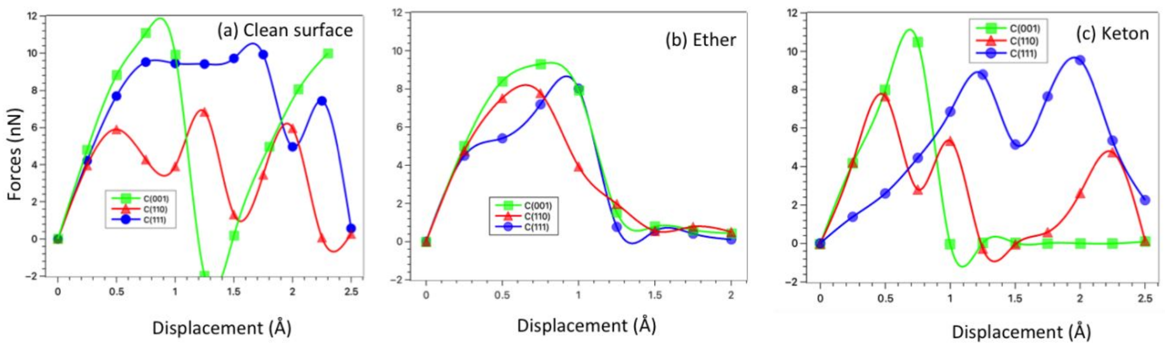

Figure S1. Forces acting on (a) carbon atom clean diamond surface, (b) oxygen atom from ether configuration, and (c) oxygen atom from Ketone configuration.

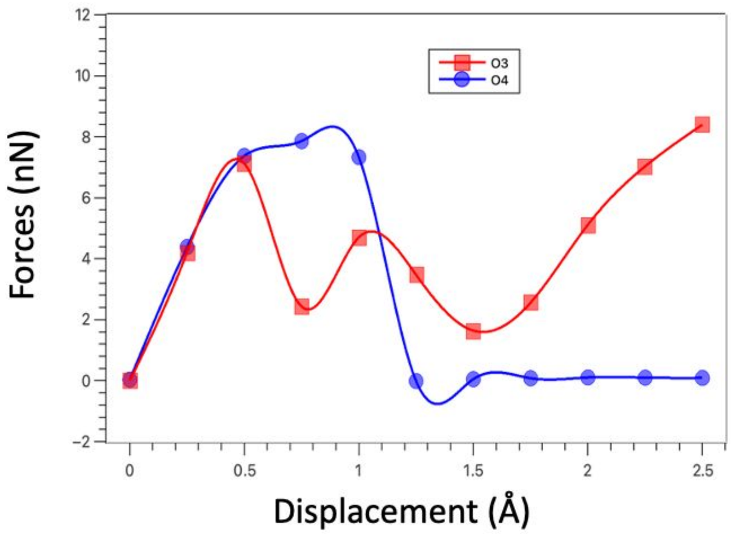

Figure S2. Forces changes during the out-of-plane displacement of the O atom belonging to the ketone group.

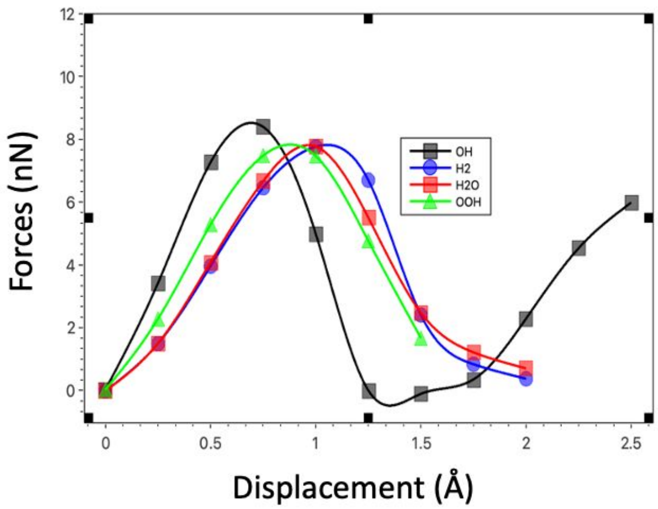

Figure S3. Forces changes during the out-of-plane displacement of the H, OH and O groups.

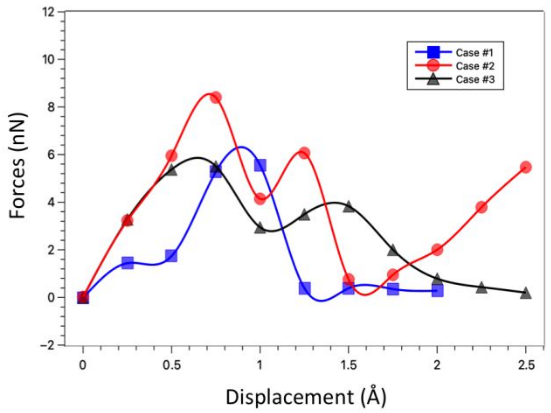

Figure S4. Force changes by the detachment of the silicate cluster on C(110) surface.

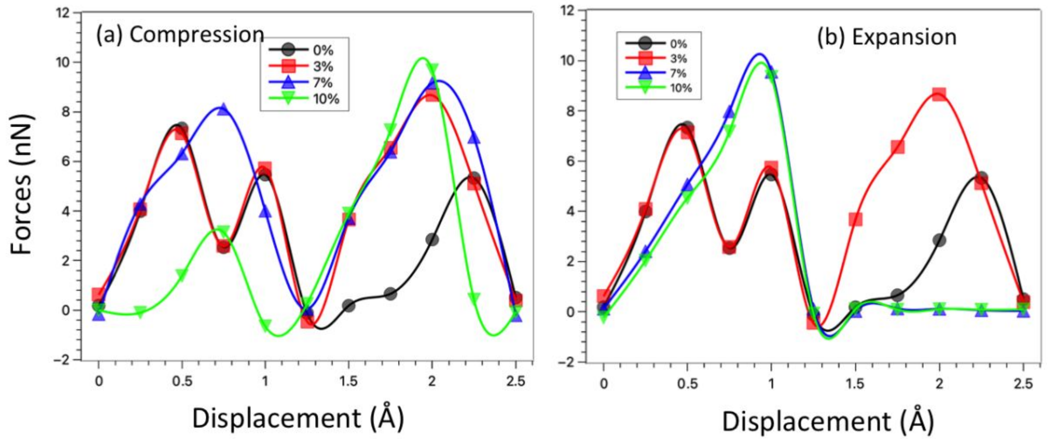

Figure S5. Force changes by the adsorption of oxygen on C(110) surface under different stress levels.
